# Use of athletic tape in the speech-language-hearing treatment of post-stroke facial paralysis in the acute phase

**DOI:** 10.1590/2317-1782/20242023153en

**Published:** 2024-05-31

**Authors:** Raquel Karoline Gonçalves Amaral, Laélia Cristina Caseiro Vicente, Tatiana Simões Chaves, Aline Mansueto Mourão

**Affiliations:** 1 Departamento de Fonoaudiologia, Universidade Federal de Minas Gerais – UFMG- Belo Horizonte (MG), Brasil.; 2 Unidade de Acidente Vascular Cerebral, Hospital Risoleta Tolentino Neves – HRTN - Belo Horizonte (MG), Brasil.

**Keywords:** Facial Paralysis, Athletic Tape, Stroke, Therapy, Speech Therapy, Facial Expression

## Abstract

**Purpose:**

To verify the efficacy of using athletic tape associated with myofunctional therapy in the speech-language-hearing treatment of facial palsy after stroke in the acute phase.

**Method:**

Randomized controlled clinical study with 88 patients with facial palsy in the acute phase of stroke. The sample was allocated in: Group 1: rehabilitation with orofacial myofunctional therapy and use of athletic tape on the paralyzed zygomaticus major and minor muscles; Group 2: rehabilitation alone with orofacial myofunctional therapy on the paralyzed face; Group 3: no speech-language-hearing intervention for facial paralysis. In the evaluation, facial expression movements were requested, and the degree of impairment was determined according to the House and Brackmann scale. Movement incompetence was obtained from measurements of the face with a digital caliper. After the evaluation, the intervention was carried out as determined for groups 1 and 2. The participants of the three groups were reassessed after 15 days. The statistical analysis used was the generalized equations.

**Results:**

The groups were homogeneous in terms of age, measure of disability and functioning, severity of neurological impairment and pre-intervention facial paralysis. Group 1 had a significant improvement in the measure from the lateral canthus to the corner of the mouth, with better results than groups 2 and 3.

**Conclusion:**

The athletic tape associated with orofacial myofunctional therapy had better results in the treatment of facial paralysis after stroke in the place where it was applied.

## INTRODUCTION

Facial palsy (FP) is one of the most common sequelae in patients affected by a stroke^(1)^, which can potentiate neurological disability with a series of negative consequences such as emotional maladjustment, psychosocial impacts, and quality of life^(2-4)^. FP can cause changes in speech, mastication, and swallowing and difficulty expressing feelings such as astonishment, joy, sadness, and anger^(2,5)^.

Some interventions in the therapeutic practice for FP aim to adapt facial symmetry at rest and in movement, rehabilitate the functions of the stomatognathic system, and restore muscle function^(5,6)^. Such interventions have shown results with immediate or lasting positive effects on therapeutic success in patients with FP in various pathologies, including stroke^(3,7,8)^.

Orofacial myofunctional therapy is one of the possibilities for the rehabilitation of facial expression, with movement-inducing massages and isotonic and isometric exercises^(3,7,9)^. This therapeutic practice can be associated with resources such as photobiomodulation, electrical stimulation, and athletic tape^(8,10)^.

Among the objectives described in the literature, athletic tape helps in the contraction and motor function of weakened muscles, in the blood and lymphatic circulation, and in favoring the increase in proprioception by exciting cutaneous mechanoreceptors^(11,12)^. Athletic tapes can promote long-lasting and constant sensory and mechanical stimuli in the skin, triggering the central nervous system and thus recruiting motor neurons, which increases muscle tone and can assist in neuroplasticity^(11,13,14)^.

The few speech-language-hearing (SLH) studies using athletic tape have already shown results with positive effects, despite their different methodologies, with and without a control group^(15-17)^.

Regarding the population with stroke, only one study has addressed the use of athletic tapes in FP in the acute phase of the disease^(8)^ but without controlling the spontaneous recovery factor. Its results demonstrated that both the group that used athletic tapes and the control group had similarly improved facial symmetry.

Thus, this study aimed to verify the effectiveness of using athletic tapes associated with myofunctional therapy in the SLH treatment of post-stroke FP in the acute phase.

## METHODS

This is a randomized controlled clinical study with a non-probabilistic sample of 88 patients with post-stroke FP in the acute phase approved by the institutional Research Ethics Committee under evaluation report no. 5.019.519. All individuals involved (or their guardians) signed an informed consent form.

The study was carried out in the stroke unit of a public hospital with patients admitted from October 2017 to September 2022. It included patients of both sexes, over 18 years old, who had a stroke in the acute phase (within 72 hours from ictus). ictus hours) and FP, conscious, alert, and understanding simple orders according to the Language Screening Test (LAST) translated into Brazilian Portuguese^(18)^. They also needed to have intact facial skin, without scars, warts, or wounds, and no medical contraindications for any procedure that was performed in this study. Individuals with craniofacial impairments other than FP, previous history of FP, comprehension deficits, degenerative diseases, and allergies to athletic tapes were excluded.

Candidates were recruited through an active search in the hospital's electronic system to find hospitalized patients, classified as ICD 10 – I64 (Stroke, not specified as hemorrhage or infarction). Hence, 150 potential participants were identified, of which 120 met the eligibility criteria and agreed to participate by signing the informed consent form. However, 22 patients were medically discharged before completing all study procedures, and 10 had a worsened clinical condition, which hindered the thorough collection. Therefore, they were excluded from the research. Thus, 88 participants completed all study procedures, most of whom were males (61.4%) and older adults (59.1%).

The sample was divided into three groups: Group 1: (n = 32) rehabilitation with orofacial myofunctional therapy and athletic tape applied on the paralyzed zygomaticus major and minor muscles; Group 2: (n = 34) rehabilitation alone with orofacial myofunctional therapy for FP; Group 3: (n = 22) without any SLH intervention for FP.

Participants were allocated to groups through stratified randomization, allowing greater homogeneity of baseline key risk factors^(19)^. Each new participant was first classified into five strata according to baseline characteristics such as age, sex, FP degree, and severity of neurological and functional impairment, and each stratum had a separate randomization list. The first research participant was assigned to Group 1, the second to Group 2, and so on.

Data were collected from patients' medical records regarding the type of stroke, head CT scan results, paralyzed side of the face, and severity of neurological impairment according to the National Institute of Health Stroke Scale (NIHSS)^(20)^, and the Functional Independence Measure (FIM)^(21)^. All participants underwent aphasia screening with LAST^(22)^ for sample selection.

Patients who met the eligibility criteria underwent initial FP assessment. They were asked to make the following facial movements to assess the respective muscles: “scared face” (occipitofrontalis muscle), “angry face” (corrugator supercilii muscle) “bad smell face” (procerus, nasalis, levator labii superioris, and ala nasi muscles), “close your eyes gently” (orbicularis oculi muscle), “close your eyes tightly” (orbicularis oculi muscle), “open smile” (zygomaticus major and minor, levator labii superioris, and levator anguli oris muscles), “closed smile” (risorius muscle), “sad face” (depressor anguli oris muscle), “lip” (mentalis muscle), and “pouching” (orbicularis oris muscle)^(6)^. The severity of FP was classified with the House and Brackmann scale^(23)^ for cases of peripheral FP, and the adapted version of the same scale as described in the literature was used to evaluate patients with central FP. Facial symmetry was assessed at rest (analyzing the midface and upper and lower face) and in movement (considering only the lower face)^(8)^.

Facial measurements were obtained with the Movement Incompetence Protocol^(24)^, using a 150-mm professional carbon fiber digital caliper, as follows: from the tragus to the corner of the mouth (T-CL), from the medial canthus to the corner of the mouth (MC-CM), from the lateral canthus to the corner of the mouth (LC-CM), and from the ala nasi to the medial canthus (AN-MC). They were measured on both sides of the participants' faces, during the open smile movement for the first three measurements and nasal contraction for the fourth measurement^(5,24)^. All measurements were taken three times, and an arithmetic mean was calculated to ensure standardization. Movement incompetence was calculated according to the formula: movement incompetence = paralyzed side – normal side x 100/normal side^(24)^.

Patients were in bed for all clinical records, sitting with support on the headrest elevated at 90º degrees, with their gaze fixed ahead, and requiring maximum effort for the movements to take the measurements. The patients were photographed and filmed in a standardized way with natural lighting, approximately 70 centimeters away from the camera – a 50 MP Infinix hot 11S mobile phone camera, 2160 p video recording, and 2480 x 1080 pix resolution. Biosafety measures were taken, such as washing hands, wearing gloves, and sanitizing the materials.

A single evaluator (the lead SLH researcher) initially assessed and reassessed the three groups every 3 days for 15 days, totaling four clinical records. This SLH pathologist has clinical experience in the management of FP and certification in the athletic tape course as recommended by the Federal SLH Council^(25)^. She was also responsible for applying the athletic tape to Group 3. The professional was not blinded during the process. However, to minimize the bias in analyzing the judgment of the degree of FP between the groups, the photos and videos of 20% of the sample with pre- and post-intervention records, without group identification, were also evaluated by another SLH pathologist who works with FP rehabilitation to analyze the interrater agreement. She did not have any previous contact with the patients participating in the study.

After the initial assessment, SLH intervention was initiated for Groups 1 and 2, whereas Group 3 was only reassessed in the set periods. It is worth mentioning that the participants in group 3 were guaranteed rehabilitation after hospital discharge, at the SLH outpatient clinic linked to the institution.

The SLH intervention was carried out through movement-inducing massages and isotonic and isometric exercises, using the dose prescribed in the SLH Intervention Protocol^(5)^. However, the treatment time was adapted as the research was carried out in a hospital, with daily 30-minute SLH follow-up, once a day, for 15 days.

All patients in groups 1 and 2 were trained to contract and maintain muscle contraction on both sides of the face while slow, deep massages were performed in the direction of muscle contraction, only on the paralyzed side. When the muscles began to show movement in the FP recovery phase, myofunctional exercises were started. The isotonic ones were quickly contracting and releasing the nose, quickly alternating closed pouching and smile, and quickly alternating open pouching and smile, all with 10 repetitions. The isometrics exercises were contracting the nose, pouching with lips closed and then open, smiles closed and then open, maintaining the movements for 5 seconds with five repetitions^(5)^. These tasks were performed with the aid of visual feedback (a mirror)^(5)^. The patient and companion were guided through training and verbal and written explanations regarding the performance of each task, three times a day, during hospitalization, being monitored once a day by the hospital's SLH team, who was trained by the lead SLH researcher and was aware of the research objectives. The SLH researcher asked the patients in all reassessments about performing the exercises and confirmed that they were performing them correctly.

For the intervention with athletic tape, patients in Group 1 used athletic tapes on the paralyzed zygomaticus major and minor muscles. A preliminary test was carried out to check whether the patient was not allergic to the tape, applying a small piece of tape to the research participant's arm, and waiting 24 hours to analyze whether there was any sign of irritation at the bonding site. After a negative response for allergy in the test, the Kinesio^®^ athletic tape (approved by the Brazilian National Health Surveillance Agency under registration 10178010268) was applied in I-shaped strips and 25% tension in the therapeutic zone – more precisely in the belly of the zygomatic muscles –, with initial anchoring on the malar surface of the zygomatic bone and final anchoring at the angle of the mouth and upper lip^(8)^, during hospital stay.

The skin area was cleaned with gauze soaked in 70% alcohol to apply the tape. Next, the researcher measured the zygomaticus major and minor muscles from origin to insertion with the measurement scale found on the back of the tape. The strips were 1.5 cm wide, and their length was defined according to each participant’s anatomical characteristics. After cutting the tape, its corners were rounded, and the therapeutic zone and anchors were marked by folding the tape. It was bonded from the insertion point to the origin of the muscles involved^(13)^.

Each participant wore the tape for 3 consecutive days; after the third day, they did not use the tape for 24 hours to rest their skin. After the skin rested, the tape was again bonded to the paralyzed muscles, following the same procedures for 15 days^(8)^.

The interrater agreement regarding the degree of FP was analyzed with the Kappa coefficient. Pre-intervention agreement was 0.809 (95% CI: 0.609 – 0.999) and post-intervention agreement was 0.929 (95% CI: 0.793 – 0.999), indicating an excellent agreement in judgments^(26)^.

The response variable of this study was the type of intervention. The explanatory variables were sex, age, side of the face affected by FP, degree of FP, neurological and functional impairment, and the value of movement incompetence.

Descriptive data analysis was performed using frequency distribution of categorical variables and measures of central tendency and variability of continuous variables. The Shapiro-Wilk test was applied for the normality of quantitative variables. Due to the normal distribution, the age of participants in the three groups was compared using analysis of variance (ANOVA) with the Bonferroni post-test, and the data were presented as means and standard deviations. NIHSS, FIM, and degree of FP, as they are qualitative variables, were compared between the three groups using the chi-square test.

The evolution of FP treatment during the acute phase of the stroke was analyzed based on pre- and post-intervention movement incompetence measurement values – which was the fourth and last reassessment record. The evolution of each group and the comparison between them were simultaneously analyzed with generalized equations. The data in this case were presented as medians and quartiles due to non-normality.

The results were obtained using IBM SPSS software, version 25, and the significance level was set at 5%.

## RESULTS

Most patients had a moderate level of neurological impairment on the NIHSS scale (63.6%) and modified dependence with assistance of up to 50% on the FIM task (59.1%). FP mostly affected the left side (68.2%), and the degree of post-stroke FP impairment ranged from moderate to total paralysis in 78.4% of cases (Table 1).

**Table 1:** Characterization of the sample

Sex		N	%
Men		54	61.4
Women		34	38.6
*Age range*			
Adults		36	40.9
Older adults		52	59.1
*Type of palsy*			
Peripheral		4	5.1
Central		84	94.9
*NIHS*			
Mild		18	20.5
Moderate		56	63.6
Severe		14	15.9
*FIM*			
Total assistance		3	3.4
Modified dependence with up to 50% assistance in the task		52	59.1
Modified dependence with up to 25% assistance in the task		15	17.0
Complete/modified independence		18	20.5
*Affected side of the face* Right		28	31.8
Left		60	68.2
*Degree of FP before the intervention*			
II – Mild dysfunction		19	21.6
III – Moderate dysfunction		28	31.8
IV – Moderately severe dysfunction		17	19.3
V – Severe dysfunction		19	21.6
VI – Total paralysis		5	5.7
*Degree of FP after the intervention*			
I – Normal		12	13.6
II - Mild dysfunction		30	34.1
III - Moderate dysfunction		18	20.5
IV - Moderately severe dysfunction		15	17.0
V – Severe dysfunction		12	13.6
VI - Total paralysis		1	1.1

**Caption:** NIHS: National Institute of Health Stroke Scale, FIM: Functional Independence Measure, FP: facial palsy

Concerning the homogeneity of the sample, the three groups did not differ regarding age (p = 0.082), measure of disability and functioning (p = 0.082), severity of neurological impairment (p = 0.165), and degree of pre-intervention FP (p = 0.963) (Table 2).

**Table 2:** Comparison of mean age, measures of disability and functioning, severity of neurological impairment, and the degree of facial palsy before the intervention between the groups

	Athletic tape + orofacial myofunctional therapy	Orofacial myofunctional therapy	No intervention	p-value
FIM				
Total assistance	0 (0%)	0 (0%)	3 (13.6%)	0.082^*^
Modified dependence with up to 50% assistance in the task	20 (62.5%)	19 (55.9%)	13 (59.1%)	
Modified dependence with up to 25% assistance in the task	5 (15.6%)	6 (17.6%)	4 (18.2%)	
Complete/modified independence	7 (21.9%)	9 (26.5%)	2 (9.1%)	
*NIHSS*				
Mild	6 (18.8%)	11 (32.4%)	1 (4.5%)	0.165*
Moderate	21 (65.6%)	18 (52.9%)	17 (77.3%)	
Severe	5 (15.6%)	5 (14.7%)	4 (18.2%)	
*Degree of facial palsy (before)*				
II – Mild dysfunction	7 (21.9%)	8 (23.5%)	4 (18.2%)	0.963^*^
III – Moderate dysfunction	11 (34.4%)	11 (32.4%)	6 (27.3%)	
IV – Moderately severe dysfunction	7 (21.9%)	5 (14.7%)	5 (22.7%)	
V – Severe dysfunction	6 (18.8%)	7 (20.6%)	6 (27.3%)	
VI – Total paralysis	1 (3.1%)	3 (8.8%)	1 (4.5%)	
*Age (years)*				
Mean (standard deviation)	65.8 (11.86)	60.6 (12.14)	67.4 (11.85)	0.082^**^

* Chi-square test;

** ANOVA

**Caption:** NIHS: National Institute of Health Stroke Scale, FIM: Functional Independence Measure.

Regarding the evolution of FP treatment during the acute phase of the stroke, all groups improved significantly in at least one measure (Table 3).

**Table 3:** Intragroup comparison of movement incompetence after the intervention

Incompetence of movement	Athletic tape + orofacial myofunctional therapy G1	Orofacial myofunctional therapy G2	No intervention G3
T-CM			
Pre-intervention	3.5 (-0.07; 11.4)	5.6 (0; 11)	9.3 (7.9; 12.1)
Post-intervention	2.7 (0.02; 8.1)	3.8 (1.8; 8.9)	7.6 (5; 9)
p-value*	0.477	0.31	**0.000**
MC-CM			
Pre-intervention	3.5 (-0.72; 9.2)	8.5 (2.8; 13.3)	6.3 (2.3; 10)
Post-intervention	0.77 (-0.08; 3.1)	3 (0.09; 7.1)	2.3 (0.08; 8.1)
p-value*	0.418	**0.006**	**0.001**
LC-CM			
Pre-intervention	6.9 (2.9; 12.2)	11 (4.1; 17.4)	9.2 (2.8; 19.4)
Post-intervention	1.4 (-0.19; 4.5)	4.3 (1.5; 7.6)	6.3 (2.5; 13.2)
p-value*	**0.006**	**0.000**	0.412
AN-MC			
Pre-intervention	0.63 (0; 5.9)	2.2 (0; 10.8)	5.6 (0; 17.8)
Post-intervention	0 (0; 1.1)	1.1 (0; 4.4)	4 (0; 14)
p-value*	0.497	0.133	0.144

* Wilcoxon test

**Caption:** T-CM: Tragus - corner of the mouth; MC-CM: medial canthus - corner of the mouth; LC-CM: lateral canthus - corner of the mouth; AN-MC: ala nasi - medial canthus

The group of patients who used athletic tape and underwent orofacial myofunctional therapy had an improvement in the LC-CM measure, precisely the one closest to the origin and insertion of the zygomatic muscles (p = 0.006). The group of patients who only underwent orofacial myofunctional therapy for FP improved in the MC-CM and LC-CM measures, and the group without intervention improved in the T-CM and MC-CM measures (Table 3).

In the intergroup comparison, the one that used athletic tape and performed orofacial myofunctional therapy had better results regarding movement incompetence than G2 and G3 in the LC-CM measure (Table 4).

**Table 4:** Intergroup comparison of movement incompetence after the intervention

Incompetence of movement	Athletic tape + orofacial myofunctional therapy G1	Orofacial myofunctional therapy G2	No intervention G3	p-value* (G1 and G2)	p-value^*^ (G1 and G3)	p-value* (G2 and G3)
T-CM	2.7 (0.02; 8.1)	3.8 (1.8; 8.9)	7.6 (5; 9)	0.579	0.19	0.359
MC-CM	0.77 (-0.08; 3.1)	3 (0.09; 7.1)	2.3 (0.08; 8.1)	0.469	0.47	0.989
LC-CM	1.4 (-0.19; 4.5)	4.3 (1.5; 7.6)	6.3 (2.5; 13.2)	**0.02**	**0.002**	0.197
AN-MC	0 (0; 1.1)	1.1 (0; 4.4)	4 (0; 14)	0.4	0.859	0.1

Kruskal-Wallis test with Bonferroni’s post-test

* Comparison between groups

**Caption:** T-CM: Tragus - corner of the mouth; MC-CM: medial canthus - corner of the mouth; LC-CM: lateral canthus - corner of the mouth; AN-MC: ala nasi - medial canthus

## DISCUSSION

The present study demonstrated that the use of athletic tape associated with orofacial myofunctional therapy had the best results in the treatment of FP in post-stroke patients in the acute phase, when compared to other study groups, in terms of incompetence of LC-CM movement – which is the measure closest to the muscles where the tape was applied.

The athletic tape proved to be an allied and promising resource for orofacial myofunctional therapy. This result reinforces the theoretical assumptions that athletic tape enhances the work of the muscle group involved in the application^(12-14)^.

According to a study with post-stroke patients with dysphagia, the elasticity of athletic tape puts a load on the rehabilitating muscle, which results in muscle activation and leads to an increase in motor unit activity in the peripheral nervous system, increasing muscle thickness^(27)^.

A systematic review used data from 12 randomized clinical trials using athletic tape to verify its kinesiological effectiveness on upper limb functions in post-stroke patients^(14)^. Patients who underwent athletic tape application had significant improvement in upper extremity function, pain intensity, shoulder subluxation, and general and flexion incapacity. However, there was no difference between groups in muscle or abduction spasticity^(14)^.

Another systematic review with meta-analysis included 18 articles to evaluate the effectiveness of athletic tape on balance function in post-stroke patients. Its results showed that athletic tape was more effective than conventional rehabilitation for balance, lower limb function, and walking^(10)^.

These studies reported an increase in sensory input, facilitating motor learning, and gaining postural adaptation strategies. Furthermore, the resource promotes the gain of functional movements by maintaining the coordination of agonist, synergist, and antagonist muscles, which corroborates the findings of the present study^(10,14)^.

It is important to highlight that the present study showed that not using athletic tape in the treatment of FP in post-stroke patients did not make it impossible to improve movement incompetence. Both the group without intervention and the group of patients who underwent only orofacial myofunctional therapy improved in some measure of movement incompetence assessed.

A study had similar findings in post-stroke patients with FP in the acute phase, also observing that both the group that underwent myofunctional therapy and application of athletic tape and the group that performed only myofunctional therapy improved facial asymmetry. There was no statistically significant difference between orofacial myofunctional therapy alone or associated with the use of athletic tape in the rehabilitation of post-stroke FP in the acute phase^(8)^. However, this study used a smaller sample than the present one, did not assess the homogeneity of the sample (which could interfere with the results of the degree of FP, age, severity of the stroke, and functioning between the groups), and did not monitor spontaneous remission in a non-intervention group^(8)^.

The literature already states that some degree of spontaneous FP remission may occur in the acute phase of stroke^(28)^, which justifies the improvement in the group without intervention. However, as observed in the results of this study and the literature already supports, spontaneous remission may not be enough to normalize facial symmetry. Hence, the patient must undergo SLH treatment early to accelerate facial nerve regeneration and avoid disordered axonal growth and muscular atrophy that indicate the sequelae phase^(6)^. Therefore, SLH therapy is recommended as early as the acute phase of FP^(6,8)^. In the present study, the clinical diagnosis of FP was early, and rehabilitation began quickly.

Orofacial myofunctional therapy in the treatment of patients with FP is essential to maintain muscle tone and readapt functional and aesthetic aspects^(9)^. Movement-inducing massages and isotonic and isometric exercises aim to recover strength and control and increase muscle mobility^(5,6)^. Therefore, the improved movement incompetence in the group that performed only orofacial myofunctional therapy is also supported by the literature^(8)^.

SLH treatment in FP aims to adapt facial symmetry at rest and in movement, rehabilitate the functions of the stomatognathic system, and recover muscular function through therapeutic practices and resources^(5,6)^. The highlight of the present study was the inter-group comparison. The group that used the athletic tape and performed orofacial myofunctional therapy had better results than the other ones in terms of the incompetence of LC-CM movement – which is the measure closest to where the zygomaticus major and minor muscles, responsible for the open smile, are located and where the athletic tape was applied. This result reinforces the hypothesis that athletic tape applied to the skin performs proprioceptive stimulation only at the site of application^(13)^.

When athletic tape is applied to the skin, it sends tactile, mechanical, proprioceptive, and kinesthetic information from the body to the somatosensory cortex, being directed to the prefrontal cortex, where all motor response planning occurs through the CNS^(11)^. Thus, the CNS receives stimuli from the athletic tape and organizes the motor response that can result in increased muscle contraction, eased pain and discomfort, improved blood circulation, corrected joint deviations, increased proprioception and relaxation, and improved tissue healing^(11,13)^.

Therefore, the use of athletic tape even in the acute post-stroke phase is an additional resource to orofacial myofunctional therapy, being an external stimulus that assists in rehabilitation, as long as the patient does not have any contraindication to its use, such as allergy^8.27^.

It is known that the treatment of FP must be based on stimulation that provides motor, visual, and proprioceptive feedback^(9)^ and that athletic tape allows patients to be aware of their movements during its use due to the stimulation of skin receptors for touch and pressure/tension^(28)^, which contributed to the G1 patients experiencing significant improvement.

A study with patients with peripheral FP used athletic tape as biofeedback for rehabilitation, and they improved faster than the control group. Moreover, athletic tape reduced the risk of muscle hypercontraction after rehabilitation, helping to improve lymphatic drainage and reduce post-exercise inflammation^(28)^.

Another study indicated its benefit as feedback in the treatment of synkinesis after Bell’s palsy and Ramsay Hunt syndrome, improving the contraction of the involuntary movement of the risorius muscle during the voluntary movement of the orbicularis oculi muscle^(29)^.

The present study aimed to verify the effectiveness of using athletic tape associated with orofacial myofunctional therapy in the treatment of post-stroke FP in the acute phase. However, one of its limitations is that no group of patients was analyzed with only athletic tape application. This would be important to verify what its isolated effects are and whether athletic tape could have a placebo effect. Furthermore, monitoring these patients after using athletic tape would also be interesting in future research to verify the lasting effect of this resource.

A study evaluated the placebo effect of athletic tape on pain and balance in individuals with postural changes, and there was a reduction in pain in participants with thoracic hyperkyphosis and shoulder protrusion; however, no beneficial effect on balance was observed. Thus, the author discusses the possible placebo effect that athletic tape has had on patients^(30)^.

Another note worth highlighting is the researchers' option to apply athletic tape to the zygomaticus major and minor muscles. These muscles were chosen because they have a greater role in the movement of an open smile. However, further studies are needed to investigate other athletic tape applications involving other facial muscles, such as the risorius, levator ala nasi, and levator labii superioris, to evaluate its effectiveness in improving movement incompetence in other facial measures.

Lastly, despite the methodological care of interrater agreement analysis of photos and videos of 20% of the sample, without group identification, to minimize the bias in the analysis of the judgment of the degree of FP between groups, triple-blind randomized clinical trials should be carried out.

The present study makes an important contribution to evidence-based practice, enabling the rehabilitation of facial expressions guided by clarity in the results. FP after stroke, still in the acute phase, must be understood as a condition that can interfere with the oral functions of the stomatognathic system and the patient's self-image, and intervention must begin in the acute phase, with the association of therapeutic resources as used in this study to improve facial expression and, therefore, functioning.

## CONCLUSION

The group of patients who used athletic tape associated with orofacial myofunctional therapy had the best results in FP treatment in post-stroke patients in the acute phase in terms of movement incompetence in the muscles where the tape was applied.

Therefore, athletic tape associated with orofacial myofunctional therapy was effective in recovering facial expression in patients in the acute phase of stroke and can be used as an auxiliary resource in SLH treatment.
