# Supplementary material for: Use of athletic tape in the speech-language-hearing treatment of post-stroke facial paralysis in the acute phase
Source: Codas. 2024 May 31;36(3):e20230153. doi: 10.1590/2317-1782/20242023153en (PMC11166036; doi:10.1590/2317-1782/20242023153en)
Supplement: Translation

# Translation: Utilização da bandagem elástica funcional no tratamento fonoaudiológico da paralisia facial pós-AVC na fase aguda

**DOI:** 10.1590/2317-1782/20242023153pt

**Authors:** Raquel Karoline Gonçalves Amaral, Laélia Cristina Caseiro Vicente, Tatiana Simões Chaves, Aline Mansueto Mourão

**Affiliations:** 1 Departamento de Fonoaudiologia, Universidade Federal de Minas Gerais – UFMG- Belo Horizonte (MG), Brasil.; 2 Unidade de Acidente Vascular Cerebral, Hospital Risoleta Tolentino Neves – HRTN - Belo Horizonte (MG), Brasil.

**Keywords:** Paralisia Facial, Bandagem Elástica, Acidente Vascular Cerebral, Reabilitação, Fonoterapia, Expressão Facial

## Abstract

**Objetivo:**

Verificar a eficácia do uso da bandagem elástica funcional associada à terapia miofuncional no tratamento fonoaudiológico da paralisia facial pós-acidente vascular cerebral na fase aguda.

**Método:**

Estudo clínico controlado randomizado com 88 pacientes com paralisia facial na fase aguda do acidente vascular cerebral. A amostra foi alocada em: Grupo 1: reabilitação com terapia miofuncional orofacial e utilização da bandagem elástica funcional nos músculos zigomáticos maior e menor paralisados; Grupo 2: reabilitação apenas com terapia miofuncional orofacial na face paralisada; Grupo 3: sem qualquer intervenção fonoaudiológica para paralisia facial. Na avaliação foram solicitados os movimentos de mímica facial e o grau do comprometimento foi determinado de acordo com a escala de House e Brackmann. A incompetência do movimento foi obtida a partir de medições da face com paquímetro digital. Após a avaliação, a intervenção foi realizada de acordo como determinado para os grupos 1 e 2. Os participantes dos três grupos foram reavaliados após 15 dias. A análise estatística utilizada foi das equações generalizadas.

**Resultados:**

Os grupos foram homogêneos quanto à idade, medida de incapacidade e funcionalidade, gravidade do comprometimento neurológico e da paralisia facial pré-intervenção. O grupo 1 teve melhora significativa na medida canto externo do olho à comissura labial, com melhores resultados quando comparado aos grupos 2 e 3.

**Conclusão:**

A bandagem elástica funcional associada a terapia miofuncional orofacial apresentou melhor resultado no tratamento da paralisia facial após acidente vascular cerebral no local onde foi aplicado.

## INTRODUÇÃO

A Paralisia Facial (PF) é uma das sequelas mais comuns em pacientes acometidos pelo Acidente Vascular Cerebral (AVC)^(1)^ podendo potencializar a incapacidade neurológica com uma série de consequências negativas como desajustes emocionais, impactos psicossociais e na qualidade de vida^(2-4)^. A PF pode ocasionar alterações na fala, mastigação e deglutição e dificuldade em expressar sentimentos como espanto, alegria, tristeza e raiva^(2,5)^.

Na prática terapêutica para PF, existem intervenções que visam adequar a simetria facial em repouso e em movimento, reabilitar as funções do sistema estomatognático e favorecer o retorno da função muscular^(5,6)^. Tais intervenções mostraram resultados com efeitos positivos imediatos ou duradouros para o sucesso terapêutico em pacientes com PF em diversas patologias, incluindo o AVC^(3,7,8)^.

A terapia miofuncional orofacial é uma das possibilidades de reabilitação da mímica facial e consiste em massagens indutoras do movimento e exercícios isotônicos e isométricos^(3,7,9)^. Essa prática terapêutica pode ser associada ao uso de recursos como fotobiomodulação, eletroestimulação e bandagem elástica funcional (BEF)^(8,10)^.

Dentre os objetivos descritos na literatura, a BEF auxilia na contração e na função motora da musculatura enfraquecida, nas circulações sanguínea e linfática e no favorecimento do aumento da propriocepção por excitação dos mecanorreceptores cutâneos^(11,12)^. A BEF pode promover estímulos sensoriais e mecânicos duradouros e constantes na pele, acionar o sistema nervoso central, ocorrendo recrutamento de neurônios motores o que resulta em aumento do tônus muscular e pode auxiliar na neuroplasticidade^(11,13,14)^.

Os poucos estudos na área da Fonoaudiologia com o uso da BEF já apontaram resultados com efeitos positivos, contudo diversas metodologias foram empregadas, com e sem grupo controle^(15-17)^.

Em relação à população com AVC, existe apenas um estudo que aborda o uso da BEF na paralisia facial na fase aguda da doença^(8)^, mas sem controlar o fator de recuperação espontânea, sendo que os resultados demonstraram tanto o grupo que utilizou a BEF como o controle apresentaram melhora da simetria facial de igual modo.

Diante do exposto, o objetivo do presente estudo foi verificar a eficácia do uso da bandagem elástica funcional associada à terapia miofuncional no tratamento fonoaudiológico da paralisia facial pós-AVC na fase aguda.

## MÉTODO

Trata-se de estudo clínico controlado randomizado com amostra não probabilística de 88 pacientes com paralisia facial pós-AVC na fase aguda aprovado pelo Comitê de Ética em Pesquisa institucional sob o parecer 5.019.519. Todos os indivíduos envolvidos (ou seus responsáveis) assinaram o Termo de Consentimento Livre e Esclarecido.

O estudo foi realizado na unidade de AVC de um hospital público com pacientes internados no período de outubro de 2017 a setembro de 2022. Foram incluídos pacientes de ambos os sexos, acima de 18 anos, que tiveram AVC, na fase aguda (em até 72 horas de ictus) e paralisia facial, conscientes, alertas, compreensão para ordens simples de acordo com *The Language Screening Test* (LAST) traduzido para o português brasileiro^(18)^. Também foi necessário estar com a pele do rosto íntegra, sem cicatrizes, verrugas ou feridas e ausência de contraindicação médica para algum procedimento que foi realizado no presente estudo. Foram excluídos os indivíduos com comprometimentos craniofaciais que diferem da paralisia facial, histórico prévio de PF, déficits de compreensão, doenças degenerativas e com alergia a bandagem elástica.

Os candidatos foram recrutados por meio de busca ativa no sistema eletrônico do hospital para encontrar os pacientes internados, classificados pelo CID 10 - I64 (Acidente Vascular Cerebral não especificado como isquêmico ou hemorrágico). Foram identificados 150 possíveis participantes, todavia 120 pacientes se enquadraram nos critérios de elegibilidade e aceitaram participar assinando o TCLE, porém 22 receberam alta médica antes da finalização de todos os procedimentos do estudo e 10 pacientes apresentaram piora do quadro clínico o que impossibilitou a coleta completa e por isso foram excluídos da pesquisa. Assim, 88 participantes concluíram todos os procedimentos do estudo, sendo a maioria do sexo masculino (61,4%) e idosos (59,1%).

A amostra foi dividida em três grupos: Grupo 1: (n= 32) reabilitação com terapia miofuncional orofacial e utilização da bandagem elástica funcional nos músculos zigomáticos maior e menor paralisados; Grupo 2: (n=34) reabilitação apenas com a terapia miofuncional orofacial para PF; Grupo 3: (n=22) sem qualquer intervenção fonoaudiológica para PF.

A alocação dos participantes nos grupos foi feita por meio de randomização estratificada, permitindo maior homogeneidade dos fatores de risco chave basais^(19)^. Cada novo participante foi primeiramente classificado em cinco estratos de acordo com as características basais como idade, sexo, grau da paralisia facial, gravidade do comprometimento neurológico e funcional e cada estrato apresentava uma lista separada de randomização. O primeiro participante da pesquisa foi designado para o grupo 1, já o segundo no grupo 2 e assim sucessivamente.

Foram coletados os dados nos prontuários dos pacientes sobre o tipo de AVC, resultados da Tomografia Computadorizada de Crânio (TCC), lado da face paralisada, gravidade do comprometimento neurológico de acordo com a *National Institute of Health Stroke Scale* (NIHSS)^(20)^ e a Medida de independência funcional (MIF)^(21)^. Todos os participantes passaram pela triagem da afasia de acordo com o LAST^(22)^ para seleção da amostra.

Os pacientes que se enquadraram nos critérios de elegibilidade, foram submetidos à avaliação inicial da paralisia facial. Os movimentos faciais solicitados e a musculatura facial avaliada foram “cara de assustado” (músculo occipitofrontal), “cara de bravo” (músculo corrugador do supercílio) “cara de cheiro ruim” (músculos prócero, nasal e elevador do lábio superior e asa do nariz), “fechar os olhos suavemente” (músculo orbicular dos olhos), “fechar os olhos com força” (músculo orbicular dos olhos), “sorriso aberto” (músculos zigomáticos maior e menor, levantador do lábio superior e levantador do ângulo da boca) , “sorriso fechado” (músculo Risório), “cara de triste” (músculo abaixador do ângulo da boca) , “beiço” (músculo mentual) e “bico” (músculo orbicular dos lábios)^(6)^. A gravidade da PF foi classificada pela escala House e Brackmann^(23)^ para os casos de paralisia facial periférica (PFP) e foi usada a versão adaptada da mesma escala conforme descrito na literatura para avaliação dos pacientes com paralisia facial central (PFC), sendo em repouso avaliada a simetria facial, analisando os três terços da face e em movimento, foi considerado apenas o terço inferior de face^(8)^.

As medidas da face foram obtidas por meio da aplicação do Protocolo de Incompetência do Movimento^(24)^ com uso do paquímetro digital profissional de fibra de carbono 150mm. As medidas foram: trágus à comissura labial (T-CL), canto interno do olho à comissura labial (CIO-CL), canto externo do olho à comissura labial (CEO-CL) e asa do nariz ao canto interno do olho (AN-CIO), mensuradas em ambos os lados da face dos participantes, durante o movimento de sorriso aberto para as três primeiras medidas e a contração nasal para a quarta medida^(5,24)^ Todas as medidas foram realizadas três vezes e foi feita uma média aritmética para garantir a padronização. A incompetência do movimento foi calculada de acordo com a fórmula: incompetência do movimento = lado paralisado – lado normal x 100/lado normal^(24)^.

Para todos os registros clínicos os pacientes se encontravam no leito, sentados com apoio na cabeceira da cama elevada a 90º graus, com o olhar fixo à frente e foi exigido esforço máximo para os movimentos solicitados durante a medição. Os pacientes foram fotografados e filmados de forma padronizada com iluminação natural do ambiente e distância de, aproximadamente, 70 centímetros, com a câmera do celular Infinix hot 11S de 50 MP, gravação de vídeo 2160 p e resolução 2480 x 1080 px. As medidas de biossegurança como, lavagem das mãos, uso de luvas e higienização dos materiais utilizados foram efetuadas.

Nos três grupos, tanto a avaliação inicial e as reavaliações, a cada 3 dias, foram realizadas por um único avaliador, fonoaudióloga pesquisadora responsável, durante 15 dias, totalizando quatro registros clínicos. Esta fonoaudióloga apresenta experiência clínica no manejo de PF e certificação no curso de bandagem conforme recomendação do Conselho Federal de Fonoaudiologia^(25)^, sendo também a profissional responsável pela aplicação da BEF no grupo 3. Não houve cegamento do profissional durante o processo, todavia, a fim de minimizar o viés de análise do julgamento do grau de paralisia facial entre os grupos, as fotos e os vídeos de 20% da amostra contendo os registros pré e pós-intervenção, sem identificação de grupo, também foram avaliadas por outra profissional fonoaudióloga que atua na área de reabilitação da paralisia facial para analisar a concordância inter-avaliadores e não teve contato prévio com os pacientes participantes do estudo.

Após a avaliação inicial foi iniciada a intervenção fonoaudiológica para os grupos 1 e 2 e para o grupo 3 foram realizadas apenas as reavaliações nos períodos estipulados. Vale mencionar, que os participantes do grupo 3 tiveram a garantia de reabilitação após alta hospitalar, no ambulatório de Fonoaudiologia vinculado à instituição.

A intervenção fonoaudiológica se deu por meio das massagens indutoras do movimento, exercícios isotônicos e isométricos e a prescrição da dose foi baseada no Protocolo de Intervenção Fonoaudiológica^(5)^ porém, o tempo de tratamento foi adaptado devido ao contexto hospitalar no qual foi realizado a pesquisa, sendo o acompanhamento fonoaudiológico diário, uma vez ao dia, por 30 minutos e com duração total 15 dias.

Todos os pacientes dos grupos 1 e 2 foram treinados a contrair e manter a contração dos músculos em ambos os lados da face enquanto eram realizadas as massagens lentas e profundas no sentido da contração muscular, apenas no lado paralisado. Quando os músculos começaram a apresentar movimento na fase de recuperação da PF foram iniciados os exercícios miofuncionais, sendo os isotônicos: contrair e soltar rapidamente o nariz, alternar rapidamente bico e sorriso fechados, alternar rapidamente bico e sorriso abertos, todos com dez repetições. Os isométricos foram: contrair o nariz, bico com os lábios fechados e depois abertos, sorrisos fechados e depois abertos, mantendo os movimentos por cinco segundos com cinco repetições^(5)^. As tarefas propostas foram realizadas com auxílio do *feedback* visual (espelho)^(5)^. O paciente e o acompanhante foram orientados por meio de treinos e explicações verbais e escritas quanto à realização de cada tarefa proposta, três vezes ao dia, durante a internação, sendo monitorados uma vez ao dia pela equipe de fonoaudiologia do hospital que foi capacitada pela fonoaudióloga pesquisadora responsável e estava ciente dos objetivos da pesquisa. Os pacientes foram indagados em todas as reavaliações pela fonoaudióloga pesquisadora quanto a realização dos exercícios e afirmaram estarem realizando corretamente.

Para a intervenção com BEF os pacientes do grupo 1 foram submetidos à utilização da bandagem elástica nos músculos zigomáticos maior e menor paralisados. Para verificar se o paciente não possuía alergia a bandagem foi realizado um teste prévio, onde foi aplicado um pedaço pequeno da fita no braço do participante da pesquisa aguardando o prazo de 24 horas para analisar se no local da colagem apresentou algum sinal de irritação. Após resposta negativa para alergia no teste, a aplicação da bandagem elástica Kinesio® Tape (aprovada pela Agência Nacional de Vigilância Sanitária sob o registro 10178010268) foi feita em tiras com formato de I e tensão de 25% na zona terapêutica, mais precisamente no ventre dos músculos zigomáticos, com a ancoragem inicial em superfície malar do osso zigomático e ancoragem final em ângulo da boca e lábio superior^(8)^, durante o período de internação do paciente.

Para aplicação da bandagem foi realizada a limpeza na região da pele com gaze embebida em álcool de 70%. Em seguida, a pesquisadora realizou a medição dos músculos zigomáticos maior e menor desde a origem até a inserção com escala de medidas que se encontra no verso da bandagem. O tamanho da bandagem na largura foi de 1,5 cm, enquanto no eixo longitudinal foi definido de acordo com as características anatômicas individuais de cada participante. Após cortar a bandagem, foi realizado o arredondamento dos cantos e a marcação da zona terapêutica e âncoras por meio da dobradura na bandagem. A colagem foi realizada do ponto de inserção para a origem dos músculos envolvidos^(13)^.

Cada participante permaneceu três dias consecutivos com a bandagem e após o terceiro dia ficou sem o uso do adesivo elástico por 24 horas para descanso da pele. Depois do período de repouso da pele, a bandagem foi novamente colada aos músculos paralisados seguindo os mesmos procedimentos durante 15 dias^(8)^.

A análise de concordância quanto ao grau da paralisia facial inter-avaliadores foi realizada por meio do coeficiente de Kappa. A concordância no pré-intervenção foi de 0,809 (IC 95%: 0,609 – 0,999) e no pós-intervenção foi de 0,929 (IC 95%: 0,793 – 0,999), indicando que houve excelente concordância nos julgamentos^(26)^.

A variável resposta deste estudo foi o tipo de intervenção. As variáveis explicativas foram: sexo, idade, lado da face acometido pela PF, grau da PF, comprometimento neurológico e funcional e o valor da incompetência do movimento.

A análise descritiva dos dados foi realizada por meio de distribuição de frequência das variáveis categóricas, medidas de tendência central e variabilidade das variáveis contínuas. O teste de Shapiro Wilk foi aplicado para a normalidade das variáveis quantitativas. Em virtude da distribuição normal, a idade dos participantes dos três grupos foi comparada por meio da análise de variância (ANOVA) com o pós teste de Bonferroni e os dados foram apresentados em forma de média e desvio padrão. As variáveis NIHSS, MIF e grau da paralisia facial, por serem variáveis qualitativas foram comparadas entre os três grupos utilizando o teste Qui Quadrado.

A evolução do tratamento na paralisia facial durante a fase aguda do AVC foi analisada a partir dos valores de medida de incompetência do movimento pré e pós-intervenção, sendo este o quarto e último registro de reavaliação. A ferramenta utilizada para analisar de maneira simultânea a evolução de cada grupo e a comparação entre eles foi das equações generalizadas (GEE). Os dados nesse caso estão apresentados em forma de mediana e quartis em virtude da não normalidade.

Os resultados foram obtidos através do *software* IBM SPSS versão 25 e o nível de significância adotado foi de 5%.

## RESULTADOS

A maioria dos pacientes tinha o nível de comprometimento neurológico moderado na escala NIHSS (63,6%) e dependência modificada com assistência em até 50% na tarefa na escala MIF (59,1%). O lado esquerdo foi o mais acometido pela paralisia facial (68,2%) e o grau de comprometimento da PF pós-AVC variou entre moderado a paralisia total em 78,4% dos casos (Tabela 1).

**Tabela 1:** Caracterização da amostra

Sexo		N	%
Homem		54	61,4
Mulher		34	38,6
*Faixa etária*			
Adulto		36	40,9
Idoso		52	59,1
*Tipo de paralisia*			
Periférica		4	5,1
Central		84	94,9
*NIHS*			
Leve		18	20,5
Moderado		56	63,6
Grave		14	15,9
*MIF*			
Assistência total		3	3,4
Dependência modificada com assistência em até 50% na tarefa		52	59,1
Dependência modificada com assistência em até 25% na tarefa		15	17,0
Independência completa /modificada		18	20,5
*Lado da face acometido* Direito		28	31,8
Esquerdo		60	68,2
*Grau da PF-pré intervenção*			
II - Disfunção leve		19	21,6
III - Disfunção moderada		28	31,8
IV - Disfunção moderadamente grave		17	19,3
V - Disfunção grave		19	21,6
VI - Paralisia total		5	5,7
*Grau da PF-pós intervenção*			
I – Normal		12	13,6
II - Disfunção leve		30	34,1
III - Disfunção moderada		18	20,5
IV - Disfunção moderadamente grave		15	17,0
V - Disfunção grave		12	13,6
VI - Paralisia total		1	1,1

**Legenda:** NIHS: *National Institute of Health Stroke Scale*, MIF: Medida de Independência funcional, PF: Paralisia facial

Em relação à homogeneidade da amostra não houve diferença na composição dos três grupos quanto à idade (p = 0,082), medida de incapacidade e funcionalidade (p = 0,082) gravidade do comprometimento neurológico (p = 0,165) e do grau da paralisia facial pré-intervenção (p = 0,963) (Tabela 2).

**Tabela 2:** Comparação da média de idade, medida de incapacidade e funcionalidade, gravidade do comprometimento neurológico e do grau da paralisia facial no momento pré-intervenção entre os grupos

	BEF + terapia miofuncional orofacial	Terapia miofuncional orofacial	Sem intervenção	valor p
MIF				
Assistência total	0 (0%)	0 (0%)	3 (13,6%)	0,082*
Dependência modificada com assistência em até 50% na tarefa	20 (62,5%)	19 (55,9%)	13 (59,1%)	
Dependência modificada com assistência em até 25% na tarefa	5 (15,6%)	6 (17,6%)	4 (18,2%)	
Independência completa /modificada	7 (21,9%)	9 (26,5%)	2 (9,1%)	
*NIHSS*				
Leve	6 (18,8%)	11 (32,4%)	1 (4,5%)	0,165*
Moderado	21 (65,6%)	18 (52,9%)	17 (77,3%)	
Grave	5 (15,6%)	5 (14,7%)	4 (18,2%)	
*Grau de paralisia facial (pré)*				
II - Disfunção leve	7 (21,9%)	8 (23,5%)	4 (18,2%)	0,963^*^
III - Disfunção moderada	11 (34,4%)	11 (32,4%)	6 (27,3%)	
IV - Disfunção moderadamente grave	7 (21,9%)	5 (14,7%)	5 (22,7%)	
V - Disfunção grave	6 (18,8%)	7 (20,6%)	6 (27,3%)	
VI - Paralisia total	1 (3,1%)	3 (8,8%)	1 (4,5%)	
*Idade (anos)*				
Média (desvio padrão)	65,8 (11,86)	60,6 (12,14)	67,4 (11,85)	0,082^**^

* Teste Qui Quadrado;

** ANOVA

**Legenda:** BEF: Bandagem Elástica Funcional NIHS: *National Institute of Health Stroke Scale*, MIF: Medida de Independência funcional

Em relação à evolução do tratamento na paralisia facial durante a fase aguda do AVC, todos os grupos apresentaram melhora significativa pelo menos em uma medida (Tabela 3).

**Tabela 3:** Comparação das incompetências do movimento intragrupos após a intervenção

Incompetência do movimento	BEF + terapia miofuncional orofacial G1	Terapia miofuncional orofacial G2	Sem intervenção G3
T/CL			
Pré intervenção	3,5 (-0,07; 11,4)	5,6 (0; 11)	9,3 (7,9; 12,1)
Pós intervenção	2,7 (0,02; 8,1)	3,8 (1,8; 8,9)	7,6 (5; 9)
valor p*	0,477	0,31	**0,000**
CIO/CL			
Pré intervenção	3,5 (-0,72; 9,2)	8,5 (2,8; 13,3)	6,3 (2,3; 10)
Pós intervenção	0,77 (-0,08; 3,1)	3 (0,09; 7,1)	2,3 (0,08; 8,1)
valor p*	0,418	**0,006**	**0,001**
CEO/CL			
Pré intervenção	6,9 (2,9; 12,2)	11 (4,1; 17,4)	9,2 (2,8; 19,4)
Pós intervenção	1,4 (-0,19; 4,5)	4,3 (1,5; 7,6)	6,3 (2,5; 13,2)
valor p*	**0,006**	**0,000**	0,412
CIO/NA			
Pré intervenção	0,63 (0; 5,9)	2,2 (0; 10,8)	5,6 (0; 17,8)
Pós intervenção	0 (0; 1,1)	1,1 (0; 4,4)	4 (0; 14)
valor p^*^	0,497	0,133	0,144

* Teste de Wilcoxon

**Legenda:** T/CL: Tragus/ Comissura labial ; CIO/CL: Canto interno do olho/ comissura labial; CEO/CL: Canto externo do olho/ Comissura labial; CIO/NA: Canto interno do olho/ asa do nariz BEF: Bandagem Elástica Funcional

O grupo de pacientes que utilizou BEF e realizou terapia miofuncional orofacial teve melhora na medida CEO-CL, justamente a medida mais próxima da origem e inserção dos músculos zigomáticos (p=0,006). O grupo de pacientes que realizou apenas a terapia miofuncional orofacial para PF teve melhora nas medidas CIO-CL e CEO-CL e o grupo sem intervenção nas medidas T-CL e CIO-CL (Tabela 3).

Na comparação intergrupos, o grupo que utilizou BEF e realizou terapia miofuncional orofacial apresentou melhores resultados quanto à incompetência do movimento quando comparado ao G2 e ao G3 na medida CEO-CL (Tabela 4).

**Tabela 4:** Comparação das incompetências do movimento intergrupos após a intervenção

Incompetência do movimento	BEF + terapia miofuncional orofacial G1	Terapia miofuncional orofacial G2	Sem intervenção G3	valor p* (G1 e G2)	valor p* (G1 e G3)	valor p^*^ (G2 e G3)
T/CL	2,7 (0,02; 8,1)	3,8 (1,8; 8,9)	7,6 (5; 9)	0,579	0,19	0,359
CIO/CL	0,77 (-0,08; 3,1)	3 (0,09; 7,1)	2,3 (0,08; 8,1)	0,469	0,47	0,989
CEO/CL	1,4 (-0,19; 4,5)	4,3 (1,5; 7,6)	6,3 (2,5; 13,2)	**0,02**	**0,002**	0,197
CIO/NA	0 (0; 1,1)	1,1 (0; 4,4)	4 (0; 14)	0,4	0,859	0,1

Teste de *Kruskal Wallis com* pós teste de *Bonferroni*

* Comparação entre grupos

**Legenda:** T/CL: Tragus/ Comissura labial ; CIO/CL: Canto interno do olho/ comissura labial; CEO/CL: Canto externo do olho/ Comissura labial; CIO/NA: Canto interno do olho/asa do nariz BEF: Bandagem Elástica Funcional.

## DISCUSSÃO

O presente estudo demonstrou que à utilização da BEF associada a realização da terapia miofuncional orofacial apresentou os melhores resultados no tratamento da PF em pacientes pós-AVC na fase aguda, quando comparada demais grupos do estudo, na medida de incompetência do movimento do canto externo do olho à comissura labial, sendo essa medida mais próxima aos músculos onde foi aplicada a bandagem.

A BEF mostrou-se um recurso aliado e promissor a terapia miofuncional orofacial. Tal resultado reforça os pressupostos teóricos de que a BEF potencializa o trabalho do grupo muscular envolvido na aplicação^(12-14)^.

De acordo com um estudo, realizado com pacientes pós-AVC com disfagia, a elasticidade da BEF fornece uma carga ao músculo que se deseja reabilitar, o que resulta em ativação muscular e leva a um aumento da atividade da unidade motora no sistema nervoso periférico proporcionando aumento da espessura muscular^(27)^.

Uma revisão sistemática utilizou dados de doze ensaios clínicos randomizados com uso da BEF, para verificar sua eficácia cinesiológica nas funções dos membros superiores em pacientes pós- AVC^(14)^. Os pacientes que realizaram a aplicação da BEF tiveram melhora significativa na função da extremidade superior, na intensidade da dor, na subluxação do ombro e na incapacidade geral e de flexão. No entanto, não houve diferença entre os grupos na espasticidade muscular ou de abdução^(14)^.

Outra revisão sistemática com meta-análise incluiu dezoito artigos para avaliar a eficácia da BEF na função de equilíbrio em paciente após-AVC e seu resultado mostrou que a BEF foi mais eficaz do que a reabilitação convencional para capacidade de equilíbrio, função dos membros inferiores e função de caminhada^(10)^.

Os estudos citados evidenciaram aumento dos *inputs* sensoriais, facilitando o aprendizado motor e o ganho de estratégias de adaptação postural. Além disso, o recurso promove o ganho de movimentos funcionais, a partir da manutenção da coordenação dos músculos agonistas, sinergistas e antagonistas, o que corrobora com os achados do atual estudo^(10,14)^.

Contudo é importante ressaltar que o presente estudo apontou que a ausência do uso da BEF no tratamento da PF em pacientes pós-AVC, não impossibilitou melhora da incompetência do movimento. Tanto o grupo sem intervenção, quanto o grupo de pacientes que realizaram apenas a terapia miofuncional orofacial obtiveram melhora em alguma medida de incompetência do movimento avaliada.

Um estudo obteve um achado similar com pacientes com PF-pós AVC na fase aguda, também observou que tanto o grupo que realizou terapia miofuncional e aplicação da BEF, quanto o grupo que realizou apenas terapia miofuncional, apresentaram melhora da assimetria da face, não havendo diferença estatisticamente significativa entre a terapia miofuncional orofacial exclusiva ou associada ao uso da BEF na reabilitação das paralisias faciais pós-AVC na fase aguda^(8)^. No entanto, esse estudo utilizou uma amostra menor quando comparada ao estudo atual, não avaliou a homogeneidade da amostra, podendo haver interferência nos resultados do grau de paralisia, idade, gravidade do AVC e funcionalidade entre os grupos e além disso, não acompanhou o fator remissão espontânea em um grupo sem intervenção^(8)^.

A literatura já preconiza que pode ocorrer algum grau de remissão espontânea da paralisia facial na fase aguda do AVC^(28)^, o que justifica a melhora do grupo sem intervenção. No entanto como foi observado nos resultados desse estudo e a literatura já defende, a remissão espontânea pode não ser suficiente para normalizar a simetria facial e o paciente deve realizar o tratamento fonoaudiológico de forma precoce a fim de acelerar o processo regenerativo do nervo facial e evitar o crescimento axonal desordenado e a atrofia muscular que indicam a fase de sequela^(6)^. Dessa forma, a atuação fonoaudiológica é preconizada ainda na fase aguda da PF^(6,8)^. No presente estudo, o diagnóstico clínico da PF foi precoce, e houve início rápido da reabilitação.

A terapia miofuncional orofacial no tratamento dos pacientes com PF é essencial para manter a tonicidade muscular e readequar os aspectos funcionais e estéticos^(9)^. As massagens indutoras do movimento e os exercícios isotônicos e isométricos são realizadas para recuperação da força, controle e aumento da mobilidade muscular^(5,6)^. Portanto, a melhora da incompetência do movimento do grupo que realizou apenas terapia miofuncional orofacial, também já é embasada pela literatura^(8)^.

O tratamento fonoaudiológico na PF visa adequar a simetria facial em repouso e em movimento, reabilitar as funções do sistema estomatognático e favorecer o retorno da função muscular por meio de práticas e recursos terapêutico^(5,6)^. O destaque do presente estudo foi na comparação inter-grupos, o grupo que utilizou o recurso terapêutico BEF e realizou a prática de terapia miofuncional orofacial apresentou melhores resultados que os outros grupos na medida de incompetência do movimento do canto externo do olho à comissura labial, sendo a medida mais próxima de onde se localizam os músculos zigomáticos maior e menor, responsáveis pelo sorriso aberto e onde foi aplicada a BEF. Tal resultado reforça a hipótese de que a BEF, aplicada sobre a pele, realiza a estimulação proprioceptiva apenas do local de aplicação^(13)^.

Quando a BEF é aplicada sobre a pele, ela envia uma informação tátil, mecânica, proprioceptiva e cinestésica do corpo, para o córtex somatossensorial, sendo direcionada para o córtex pré-frontal, onde ocorre todo o planejamento da resposta motora pelo SNC^(11)^. Desta forma, o SNC recebe estímulos provenientes da BEF e organiza a resposta motora que pode resultar em aumento da contração muscular, diminuição da dor e da sensação de desconforto, melhora da circulação sanguínea, correções de desvios articulares, aumento da propriocepção, relaxamento e melhorar a cicatrização tecidual^(11,13)^.

Com isso, o uso da BEF ainda na fase aguda pós-AVC é um recurso adicional à terapia miofuncional orofacial, sendo um estímulo externo que auxilia na reabilitação, desde que o paciente não tenha qualquer contraindicação para o seu uso como por exemplo, alergia. ^8.27^.

Sabe-se que o tratamento da PF deve ser pautado em estimulações que proporcionem *feedbacks* motores, visuais e proprioceptivos^(9)^ e que a BEF proporciona que os pacientes fiquem cientes de seus movimentos durante o seu uso devido à estimulação dos receptores da pele para toque e pressão/tensão^(28)^ o que contribuiu para que os pacientes do G1 tivessem melhora significante.

Ademais, um estudo realizado com paciente com PFP utilizou a BEF como *biofeedback* para reabilitação e esses pacientes obtiveram melhora mais rápida em relação ao grupo controle, além da BEF reduzir o risco de hipercontratura dos músculos após a reabilitação, ajudar a melhorar a drenagem linfática e reduzir a inflamação pós-exercício^(28)^.

Outro estudo indicou o seu benefício como *feedback* no tratamento das sincinesias pós-PF de Bell e síndrome de Ramsay Hunt apresentando melhora da contração do movimento involuntário do músculo risório durante a movimentação voluntária do músculo orbicular do olho^(29)^.

Apesar de o objetivo do presente estudo ter sido verificar a eficácia do uso da bandagem elástica funcional associada à terapia miofuncional orofacial no tratamento da paralisia facial pós-AVC na fase aguda, como limitação pode-se considerar que não foi analisado um grupo de pacientes com somente aplicação da BEF. Isso seria importante para avaliar quais são seus efeitos isolados e se a BEF poderia ter um efeito placebo. Ademais, o acompanhamento desses pacientes após o uso da BEF também seria interessante, em pesquisas futuras, a fim de verificar o efeito duradouro desse recurso.

Um estudo avaliou o efeito placebo da BEF na dor e no equilíbrio de indivíduos com alteração postural e houve redução na dor dos participantes com hipercifose torácica e protrusão de ombros, no entanto, não foi observado efeito benéfico no equilíbrio. Dessa forma, o autor discute sobre o possível efeito placebo que a BEF tenha tido nos pacientes^(30)^.

Outro apontamento que vale destaque é a opção dos pesquisadores na aplicação da BEF nos músculos zigomático maior e menor. Esses músculos foram escolhidos por terem maior função no movimento do sorriso aberto, porém, são necessários estudos que investiguem novas aplicações da BEF que envolvam outros músculos da face, como por exemplo risório e elevador da asa do nariz e do lábio superior para avaliar a sua eficácia na melhora da incompetência do movimento nas demais medidas da face.

Por fim, apesar do cuidado metodológico de análise de concordância inter-avaliadores de fotos e vídeos de 20% da amostra, sem identificação de grupo, a fim de minimizar o viés de análise do julgamento do grau de paralisia facial entre os grupos, sugere-se estudos de ensaio clínico randomizado triplo-cego.

O presente estudo traz contribuição importante para a prática baseada em evidência, propiciando que a reabilitação da mímica facial seja pautada por clareza nos resultados obtidos. A PF após o AVC ainda na fase aguda, deve ser compreendida como um comprometimento que pode interferir nas funções orais do sistema estomatognático e na autoimagem do paciente, devendo-se iniciar a intervenção ainda na fase aguda, com associação de recurso terapêuticos como utilizado neste estudo, a fim de promover a melhora da mímica facial e consequentemente, a funcionalidade.

## CONCLUSÃO

O grupo de pacientes que utilizou a BEF associada à terapia miofuncional orofacial apresentou os melhores resultados no tratamento da PF em pacientes pós-AVC na fase aguda na medida de incompetência do movimento nos músculos onde foi aplicada a bandagem.

Dessa forma, a BEF associada à terapia miofuncional orofacial foi eficaz para a recuperação da mímica facial em pacientes na fase aguda do AVC e pode ser utilizada como recurso coadjuvante no tratamento fonoaudiológico.
